# Stable Transfection of *Eimeria intestinalis* and Investigation of Its Life Cycle, Reproduction and Immunogenicity

**DOI:** 10.3389/fmicb.2016.00807

**Published:** 2016-05-31

**Authors:** Tuanyuan Shi, Geru Tao, Guolian Bao, Jingxia Suo, Lili Hao, Yuan Fu, Xun Suo

**Affiliations:** ^1^Department of Animal Parasitology, Institute of Animal Husbandry and Veterinary Medicine, Zhejiang Academy of Agricultural ScienceHangzhou, China; ^2^National Animal Protozoa Laboratory, College of Veterinary Medicine, China Agricultural UniversityBeijing, China; ^3^College of Life Science and Technology, Southwest University for NationalitiesChengdu, China

**Keywords:** *Eimeria intestinalis*, YFP, stable transfection, life cycle, reproduction, pathogenicity

## Abstract

Rabbit coccidiosis, caused by infection of *Eimeria* spp. is one of the most severe parasitic diseases in rabbits. *Eimeria intestinalis* is one of the most immunogenic species in rabbit coccidia. Due to the lack of genomic information and unsuccessful *in vitro* cultivation, genetic manipulation of rabbit coccidia lagged behind other apicomplexan parasites. Using regulatory sequences from *E. tenella*, we obtained a transgenic line of *E. intestinalis* expressing yellow fluorescent protein (YFP). YFP was continuously expressed throughout the whole life cycle. Morphological features of *E. intestinalis* in different developmental stages were dynamically observed with the transgenic line. Some important features in the endogenous development stages were observed. Trophozoites were found as early as 4 h post inoculation. Two types of schizonts and merozoites were observed in first three of the four schizogonies. Beside jejunum and ileum, gametogony stage and oocysts were also found in the duodenum and vermiform appendix. In addition, the transgenic strain was highly immunogenic but less pathogenic than the wild type. Considering the high immunogenicity of *E. intestinalis* and amenability to transfection with foreign genes, transgenic *E. intestinalis* could be a promising oral eukaryotic vaccine vector.

## Introduction

Coccidiosis is one of the most common and highly contagious diseases in rabbits, causing severe economic losses in rabbitries due to reduced weight gain and high mortality. Currently, treatment with anticoccidial drugs is the only effective measure in control of rabbit coccidiosis (Pakandl, 2009). However, vaccination with live oocysts is the preferred method since the use of anticoccidial drugs is associated with the emergence of drug resistance strains and drug residues in rabbit meat products.

Genetic manipulation is a powerful technique to study parasite biology and immunology, and to develop novel vaccines against infectious diseases. Transfection of apicomplexan parasites has transformed the studies of this important group of pathogens. By disruption of the locus or replacement with an altered or tagged gene, functions of specific genes have been studied for the apicomplexan parasites *Toxoplasma gondii* and *Plasmodium* spp. (Goodewardene et al., 1993; Donald and Roos, 1993; Soldati and Boothroyd, 1993; Sibley et al., 1994; de Koning-Ward et al., 2000; Li et al., 2015a). However, research advances on transfection of *Eimeria*, which are also of the apicomplexan family, are slower than those of *T. gondii* and *Plasmodium* spp. This is mainly because *Eimeria* spp. cannot be continuously cultivated *in vitro*. Stable transfection of *Eimeria* requires preparation and purification of sporozoites from oocysts, inoculation of transfected sporozoites into parasitic sites of host animals and propagation of selected oocysts. Fortunately, stable transfection systems have been successfully established in a rat Eimerian species, *Eimeria nieschulzi* (Kurth and Entzeroth, 2009), and two chicken Eimerian species, *E. tenella* (Yan et al., 2009) and *E. mitis* (Qin et al., 2014).

Eimerian parasites can stimulate strong antibody and cell-mediated immunity (Wallach, 2010). Their exceptional potential as vectors delivering heterologous antigens to the mucosal immune system were well recognized (Shi et al., 2008; Yan et al., 2009; Clark et al., 2012). Two transgenic lines of *E. tenella* were reported to induce exogenous antigen-specific immune responses (Huang et al., 2011; Clark et al., 2012), and another transgenic *E. mitis* expressing chicken interleukin 2 strongly enhanced cellular immunity against wild type parasite challenge (Li et al., 2015b). However, little information was available on the transfection of rabbit Eimerian species.

In this study, we, for the first time, established a stable transfection system in a rabbit coccidian species, *E. intestinalis*, which is one of the most immunogenic coccidia species (Licois et al., 1990; Coudert et al., 1993, 1995). Taking advantages of the fluorescence, we identified important features in the endogenous development stages of this parasite. Moreover, this transgenic line showed reduced pathogenicity but maintained high immunogenicity, indicating it could be developed as an antigen delivery vector.

## Materials and Methods

### Parasites, Animals, and Cell Culture

A low-virulent *E. intestinalis* strain, which was isolated and identified by Shi et al. (2014) was used in the study. About 5 × 10^7^ freshly sporulated oocysts were first ground to release sporocysts. Then sporocysts were excysted in a digestion solution (PBS pH7.4, containing 10% chicken bile and 0.75% trypsin). Released sporozoites were purified by a DE-52 cellulose column.

Coccidia-free rabbits were reared in a coccidia-free environment in our institute according to the modified therapeutic method described by Coudert et al. (1995). The young rabbits were weaned at the age of 18 days, and then fed human infant formula (Beingmate, China) in combination with rabbit pellets (prepared in our institute) till 30 days old. Feed and water were sterilized and provided *ad libitum*. Feces was checked daily for any coccidian oocysts.

Primary rabbit small intestine cells (PRICs) were obtained from newborn rabbits and used for transient transfection of *E. intestinalis*. Newborn rabbit pups prior to their first suckling were euthanized and then immersed in 75% ethanol for 10 min to kill external microorganisms. The small intestine including distal duodenum, jejunum and ileum were excised and longitudinally opened, and intestinal contents were removed. The intestinal wall of 0.5 cm^2^ was washed with sterilized PBS and then digested in collagenase IV solution at 37°C for 30 min. PRICs were isolated by centrifugation at 1000 rpm for 5 min, and grown in the RPMI-1640 culture medium supplemented with fetal bovine serum [10% (v/v)], penicillin (200 U/ml), and streptomycin (20 mg/ml) at 39.5°C in an atmosphere of 5% CO_2_.

Our research with animals was approved by the Beijing Administration Committee of Laboratory Animals and performed in accordance with the China Agricultural University Institutional Animal Care and Use Committee guidelines.

### Plasmid and Parasite Transfection

The plasmid *p*EtHEA, constructed in Professor Xun Suo’s laboratory, was used to transfect *E. intestinalis* (Yan et al., 2009). The plasmid carries a 1090 bp promoter from the 5′ flanking sequence of *E. tenella* histone 4 gene (H4), a 1467 bp sequence of tandem yellow fluorescent protein (YFP) gene (YFP–YFP), and a 1402 bp terminator from the 3′ untranslated region sequence of *E. tenella* actin gene (Actin). Transfection of *E. intestinalis* sporozoites with *p*EtHEA was carried out according to the method of Liu et al. (2008). Electroporation was performed using a Gene Pulser X Cell^TM^ (Bio-Rad, USA) at 2000 v and 25 μF with a pulse time of about 0.3 ms. After electroporation, the parasites were left undisturbed at room temperature for at least 20 min, and then transferred to a confluent monolayer of PRICs. Transfected sporozoites were examined under a fluorescent microscope (Olympus, Japan) 24 h post inoculation (pi).

For stable transfection, approximately 1 × 10^6^ transfected sporozoites were surgically inoculated into the duodenum of a coccidia-free rabbit. Oocysts from feces excreted between 9 and 14 days pi were collected using the saturated NaCl flotation method and sporulated in 2.5% potassium dichromate at 28°C.

Sporulated oocysts were purified with 13% sodium hypochlorite solution and then washed with sterilized PBS. Transgenic oocysts were screened by fluorescence- activated cell sorting with a MoFlo Cell Sorter (Dako Cytomation, Fort Collins, CO, USA). Sorted fluorescent oocysts were propagated in coccidia-free rabbits. Stably transfected *E. intestinalis* were obtained after five cycles of sorting and propagation in coccidia-free rabbits.

### Observation of the Life Cycle of Transgenic *E. intestinalis*

To study the life cycle of the transgenic *E. intestinalis*, five rabbits were inoculated with 1 × 10^6^ sporulated oocysts each for observation of early endogenous development stages and four rabbits were inoculated with 1 × 10^3^ sporulated oocysts each for observation of late endogenous developmental stages. Intestine smears were made by scraping the mucosal membrane of duodenum, jejunum, ileum, cecum, vermiform appendix, and colon of the rabbits euthanized 4, 24, 48, 72, 96, 120, 168, 216, and 336 h pi. For observation of sporogony of the parasites, freshly excreted oocysts were collected at 336 h pi from fecal samples, and allowed to sporulate in 2.5% potassium dichromate solution. Observation of the parasites was conducted under a fluorescence microscope.

### Determination of Reproduction and Immunogenicity of the Transgenic Parasites

Sixteen 34-day-old coccidia-free rabbits housed 2 per cage were distributed into four groups with four rabbits per group. One group was orally inoculated with 5 × 10^3^ sporulated oocysts of *E. intestinalis* wild type. Same amount of oocysts of *E. intestinalis* transgenic strain were inoculated to another group. Both groups and a non-immunized, challenged control group were orally challenged with 1 × 10^6^ sporulated oocysts of the wild type parasites 16 days pi. The last group was not immunized or challenged and served as a negative control.

Body weights of all rabbits were measured at 0 and 16 days after immunization and at 10 and 19 days after challenge. Feces were collected daily from 9 to 15 days after immunization and 9 to 16 days after challenge. Total oocyst output in feces were counted in a McMaster chamber.

### Statistical Analysis

Statistical analysis was performed by one-way ANOVA using the SPSS 16.0 software. Data were expressed as mean ± standard deviation. Differences between groups were considered statistically significant when *p*-values were less than 0.05.

## Results

### Transient Expression of YFP in *E. intestinalis*

**FIGURE 1:**
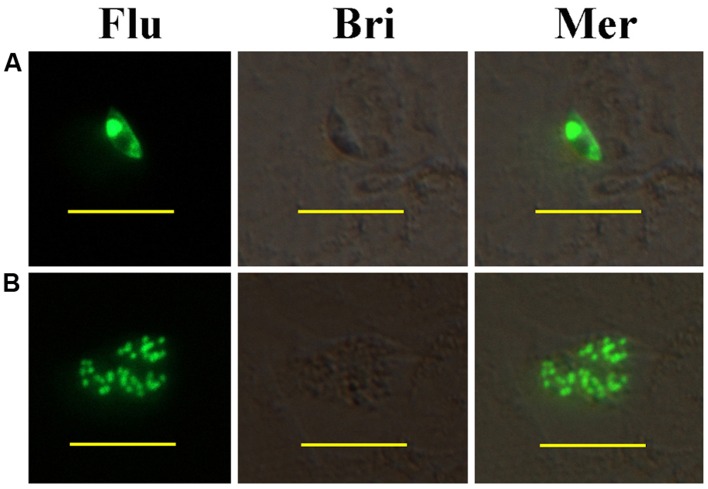
**Development of transiently transfected *Eimeria intestinalis* sporozoites in primary rabbit intestinal cells (PRICs). (A)** A sporozoite expressing YFP in PRICs; **(B)** a first generation schizont expressing YFP in PRICs. Flu, Bri, and Mer indicate fluorescent field, bright field, and merged images, respectively. Bar = 20 μm.

*In vitro*, fluorescent sporozoites and first generation schizonts were observed after inoculation to PRICs. YFP was mainly located within nucleus of the parasites (**Figures 1A,B**). No fluorescence was observed in the refractile body or parasitophorous vacuole. This localization was determined by a nucleus-targeting signal sequence incorporated in the histone 4 promoter of *p*EtHEA.

### Stable Transfected *E. intestinalis* and Its Development in the Whole Life Cycle

First, we obtained a stable transgenic line of *E. intestinalis.* Fluorescent oocysts were observed from rabbit feces on the 9th day after injection of transfected sporozoites. Microscopic observation and cytometry analysis revealed that only 0.01% of the first generation expressed YFP (data not shown). However, in subsequent passages, fluorescent rate increased to 21.64% in the second generation and 80% in the third generation. Similar results with the same plasmid had been reported in *E. tenella* by Yan et al. (2009), where the percentage of fluorescent oocysts was only 0.04% in the first generation and rose to 87.5% in the third generation. Collectively the findings suggested that the constructed sequence of *p*EtHEA is conserved among the genus *Eimeria* and it is highly functional and efficient in *E. intestinalis*.

**FIGURE 2:**
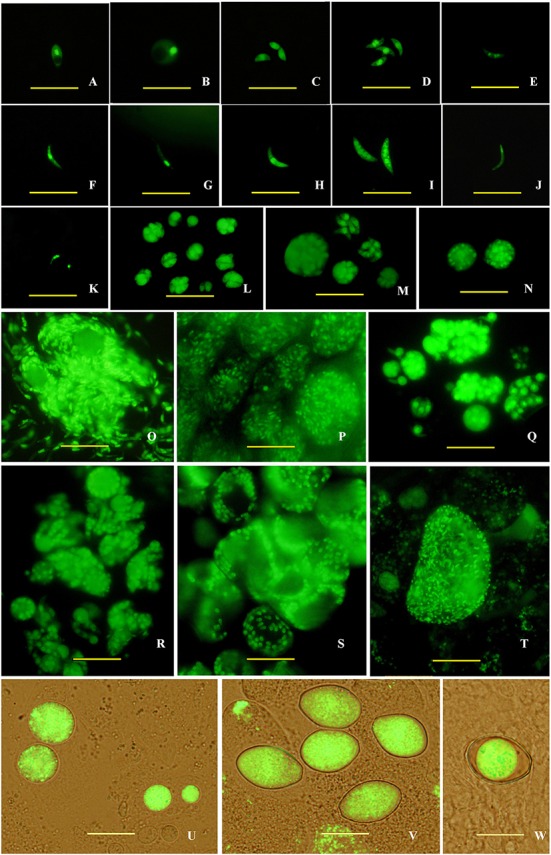
**Endogenous development of the transgenic *E. intestinalis* expressing YFP in rabbits. (A)** A sporozoite at 4 h pi; **(B)** a trophozoite at 4 h pi; **(C,D)** first generation merozoites at 96 h pi; **(E–G)** second generation merozoites at 120 h pi; **(H,I)** third generation merozoites at 168 h pi; **(J)** a fourth generation merozoite at 216 h pi; **(K)** two microgametes at 216 h pi, **(L,M)** first generation schizonts at 96 h pi; **(N–P)** second generation schizonts at 120 h pi; **(Q,R)** third generation schizonts at 168 h pi; **(S)** fourth generation schizonts at 216 h pi; **(T)** a microgamont at 216 h pi; **(U)** macrogamonts at 216 h pi; **(V)** Zygotes at 216 h pi; **(W)** a mature oocyst at 216 h pi. Bar = 20 μm.

Then, endogenous development stages of transgenic *E. intestinalis* in rabbit intestines were observed under a fluorescent microscope between 4 and 216 h pi. Sporozoites mainly parasitized in the lower jejunum and ileum (**Figure 2A**), and developed into trophozoites as early as 4 h pi. These trophozoites were mononuclear and appeared spherical (**Figure 2B**). They developed into first generation schizonts containing several, usually less than five merozoites (**Figures 2L,M**) at 72 h pi. The first generation merozoites were the smallest in four schizogony stages and released at 96 h pi. Two forms of merozoites were observed: one appeared ovoid (**Figure 2C**), and the other was boat-shaped with two pointy ends and were slightly longer than the ovoid merozoites (**Figure 2D**). They all contained one large nucleus located in the middle region of the parasite. Large second generation schizonts containing dozens to hundreds of merozoites (**Figures 2N–P**) were found at 120 h pi. The second generation merozoites were also of two types. One was multinuclear and crescent-shaped (**Figure 2E**), and the other type was uninucleate, banana-shaped and longer than the multinuclear merozoites (**Figures 2F,G**). Both types of second generation merozoites were more slender than first generation merozoites. The third generation schizonts were observed at 168 h pi, housed several to dozens of merozoites (**Figures 2Q,R**) and were smaller than the second generation schizonts. Both two types of the third generation merozoites were the largest of the four schizogony stages. The uninucleate merozoites (**Figure 2H**) was smaller than multinuclear types (usually 4 to 5 nuclei; **Figure 2I**). There was only one form of Fourth generation schizonts, which contained dozens of uninucleate merozoites and were found at 216 h pi. The nucleus was located in the anterior end of the merozoites (**Figure 2J**). A large vesicle was observed in the meronts (**Figure 2S**).

**FIGURE 3:**
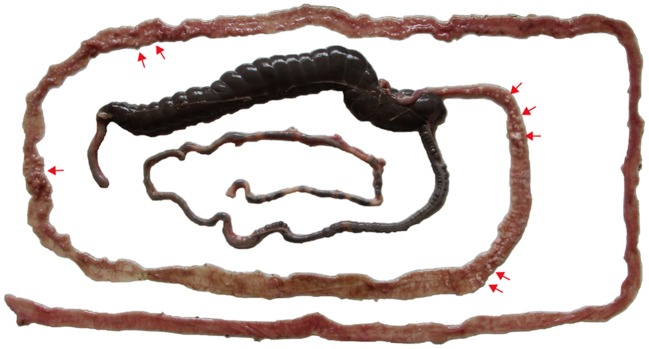
**Pathological changes of white nodules (red arrows) of the lower jejunum and ileum of a rabbit at 9 days after inoculation with 5 × 10^3^ transgenic *E. intestinalis* oocysts**.

Parasites in the gametogony stage (including macrogametes, microgamonts, microgametes, and zygotes) and oocysts were mainly found in the lower jejunum and ileum at 216 h pi, but also found in duodenum and vermiform appendix. Microgamonts contained thousands of microgametes (**Figure 2T**), which were tadpole- shaped with one nucleus located in the anterior end (**Figure 2K**). Macrogametes appeared spherical or elliptical (**Figure 2U**). Zygotes and oocysts all appeared piriform (**Figure 2V**), but the oocyst wall was obvious in mature oocysts and the cytoplasm was usually condensed into a sphere (**Figure 2W**). In addition, nodule formation and small intestine wall thickening were found in the lower jejunum and ileum after 9 days pi (**Figure 3**).

**FIGURE 4:**
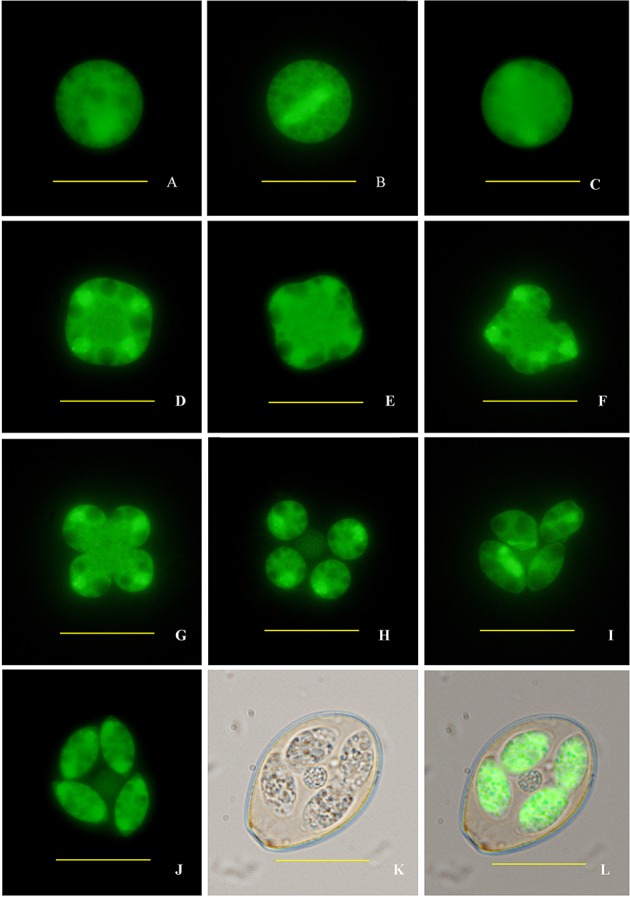
**Sporogony of transgenic *E. intestinalis* expressing YFP. (A)** An unsporulated oocyst; **(B)** spindle stages showing spindle projections; **(C)** first nuclear division complete; **(D)** second nuclear division complete; **(E)** early cytokinesis; **(F,G)** late cytokinesis; **(H,I)** sporoblasts; **(J–L)** a sporulated oocyst (fluorescent, bright, and merged images, respectively). Bar = 20 μm.

Finally, sporogony of transgenic oocysts in 2.5% potassium dichromate solution at 28°C was observed by fluorescence microscopy. The sporulation process encompassed three times of nuclear division and two times of cytokinesis, and completed within 30 h (**Figure 4**). After the second nuclear division (**Figures 4A–C**), four nuclei formed and the cell body became quadrilateral (**Figures 4D,E**). Then four sporoblasts symmetrically formed in four directions. After the first cytokinesis, these concave grooves separated from the mother cell into four symmetric daughter cells (**Figures 4F–H**). At the same time, oocyst residual body formed during the second nuclear division after releasing from the mother cell (**Figure 4H**). Two sporozoites formed in each daughter cell after the third nuclear division and the second cytokinesis (**Figures 4G–L**). Each sporozoite contained two refractile bodies of different sizes, which began to form before the second nuclear division and were not fluorescent under the fluorescence microscope (**Figure 4I**).

### Reproductivity and Immunogenicity of the Transgenic *E. intestinalis*

First, oocyst outputs per rabbit inoculated with both strains were determined (**Table 1**). Oocyst yield of the transgenic *E. intestinalis* was significantly less than that of the wild type (by ~87%; *p* ≤ 0.05), indicating a weakened reproductivity of transgenic *E. intestinalis*.

**Table 1:** Oocyst output in rabbits (mean ± SD; *n* = 4) after immunization with sporulated wild type or transgenic oocysts (5 × 10^3^) and challenged with the wild type oocysts (1 × 10^6^) of *Eimeria intestinalis.*

Immunization	Challenge	Oocyst output per rabbit after immunization	Oocyst output per rabbit after challenge	Reduced percentage of oocyst output with the challenge control group
Wild type oocysts	Wild type oocysts	19.22 × 10^8^ ± 2.069 × 10^8^	2.81 × 10^8^ ± 0.235 × 10^8^	84.35%
Transgenic oocysts	Wild type oocysts	2.57 × 10^8^ ± 0.333 × 10^8^	6.79 × 10^8^ ± 0.356 × 10^8^	62.25%
None	Wild type oocysts	0	18.09 × 10^8^ ± 1.815 × 10^8^	–
None	None	0	0	–

**FIGURE 5:**
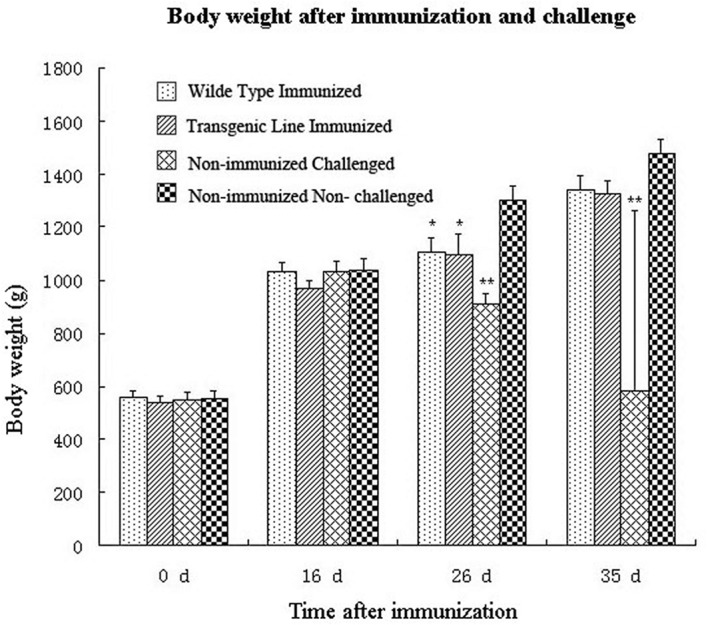
**Body weights of rabbits after immunization with 5 × 10^3^ transgenic or wild-type *E. intestinalis* oocysts and challenged with 1 × 10^6^ wild-type *E. intestinalis* oocysts at 16 days after immunization (*n* = 4).** ^∗^*p* < 0.05, ^∗∗^*p* < 0.01 (compared with the unimmunized and unchallenged group).

Second, rabbits were challenged with a high dose of wild type *E. intestinalis* oocysts to determine the immunogenicity of the transgenic parasites. No case of diarrhea or mortality was seen after challenge in immunized rabbits. Although body weights of immunized rabbits were lower than that of the non-immunized non-challenged group, there was no significant difference between the two immunized groups (*p* > 0.05; **Figure 5**). Body weight of the non-immunized, challenged group was significantly lower than all other groups. In addition, similar oocyst reductions were detected in the wild type and transgenic parasites immunized groups after challenge (**Table 1**),indicating that the transgenic *E. intestinalis* possessed similar immunogenicity and protective efficacy to the wild type parasites against homologous parasite infection.

## Discussion

In our study, a stable transfection system was successfully established in *E. intestinalis*. This is the first report on transgenesis of rabbit coccidia and an important progress in genetic manipulation of Eimerian parasites.

The plasmid used in our study contained regulatory sequences originating from *E. tenella*. Another study on transfection of *E. mitis* was conducted using similar method, as well (Qin et al., 2014). Regulatory sequences of *E. tenella* housekeeping genes are highly conserved and functional among *Eimeria* spp., which is valuable in studying other *Eimeria* spp. since only a few species including *E. tenella* have been genomically mapped (Shirley, 2000; Chapman et al., 2013; Blake, 2015). Stably transfected rabbit *Eimeria* spp. had not been previously achieved because it was time- consuming to rear coccidia-free rabbits, *E. intestinalis* sporozoites are more difficult to purify than *E. tenella*, and inoculation of transfected sporozoites into the small intestine of rabbits requires abdominal surgery.

In our transgenic *E. intestinalis*, the fluorescent protein was expressed mainly in the nuclei of the parasites. It is valuable for the identification of parasite in different developmental stages. In a previous study on transgenic *E. mitis*, a pair of flagella of microgametes was newly identified (Qin et al., 2014). In our study, apart from major features of parasite development consistent with earlier reports, we found some important features which have not been previously described. Trophozoites were detected as early as 4 h pi, which was considerably sooner than 24–48 h pi. reported previously (Licois et al., 1992; Yin et al., 1993). Besides lower jejunum and ileum, parasites of the gametogony stage were also found in duodenum and vermiform appendix.

The expression level of exogenous protein is generally low in transgenic *Eimeria* spp. Because of the low foreign protein expression and low dose of virulent *Eimeria* spp. that can be safely administered as a vaccine vector, vaccination using virulent strains of *Eimeria* spp. would induce low exogenous protein-specific immune response. Thus, low virulent yet immunogenic strains as vaccine vectors, for instance, the transgenic *E. intestinalis* developed by us may be able to stimulate robust immunity without adversely affecting animal growth and meat production.

## Author Contributions

TS and XS designed this study. TS carried out the experiments with the help of GT, GB, JS, LH, and YF. XS supervised the study implementation. TS drafted the manuscript. GT and XS contributed the revision of the manuscript. All authors read and approved the final version of the manuscript.

## Conflict of Interest Statement

The authors declare that the research was conducted in the absence of any commercial or financial relationships that could be construed as a potential conflict of interest.
